# Joint Canadian Association of Gastroenterology and Crohn’s Colitis Canada Position Statement on Biosimilars for the Treatment of Inflammatory Bowel Disease

**DOI:** 10.1093/jcag/gwz035

**Published:** 2019-11-08

**Authors:** Paul Moayyedi, Eric I Benchimol, David Armstrong, Cathy Yuan, Aida Fernandes, Grigorios I Leontiadis

**Affiliations:** 1 Division of Gastroenterology and Farncombe Family Digestive Health Research Institute, McMaster University, Hamilton, Ontario, Canada; 2 Children’s Hospital of Eastern Ontario (CHEO) Inflammatory Bowel Disease Centre, Division of Gastroenterology, Hepatology and Nutrition and CHEO Research Institute, Ottawa, Ontario, Canada; 3 Department of Pediatrics and School of Epidemiology and Public Health, University of Ottawa, Ottawa, Ontario, Canada

**Keywords:** Biosimilars, Canadian Association of Gastroenterology, Crohn’s Colitis Canada, Inflammatory bowel disease

## Introduction

The development of anti-tumour necrosis factor (TNF) therapies has transformed the care of patients with immune-mediated diseases such as inflammatory bowel disease (IBD), including ulcerative colitis and Crohn’s disease, psoriasis and inflammatory arthropathies, including rheumatoid arthritis and ankylosing spondylitis. For IBD patients, these biologic therapies are effective at inducing and maintaining remission (1), reducing the need for surgery (2) and improving quality of life (3). Although anti-TNF therapies are effective in several different immune-mediated disorders, individual biologics are not, necessarily, equally effective in all disorders suggesting that their actions may differ between disorders. For example, the paradox that anti-TNF drugs used to treat rheumatoid arthritis (RA) and psoriasis may yet cause joint pains and skin reactions in IBD patients, emphasizes our imperfect understanding of the mechanism of action of these biologic therapies and the need to evaluate treatment outcomes separately for different disorders.

The main factors that limit the use of anti-TNF drugs are adverse events and cost. Anti-TNF therapy is associated with joint pains, dermatological disorders and transfusion reactions (4). Transfusion reactions are associated with the development of antibodies to anti-TNF agents and this may also be associated with reduced treatment efficacy (5). Anti-TNF agents are also associated with an increased risk of infection (6) and risk of lymphoma although these risks may be exacerbated, to a greater or lesser extent, by the concomitant use of azathioprine or other immunosuppressive agents (7).

Anti-TNF therapies are also expensive costing up to $20,000 per year for each patient in Canada. Canada spent more on biologic therapies for all indications than on any other class of drug in 2018 accounting for 8.2% of the $33.7 billion spent on prescription medications (8). While the cost of these therapies is significant, the cost of having IBD is also expensive to society. It is estimated that the indirect cost of IBD was $1.29 billion in Canada in 2018 (9,10). Anti-TNF therapies can improve quality of life and productivity and, accounting for these societal costs, biologics may offer value for money (11). In the Canadian setting, health care is mainly funded centrally by the tax payer and the government, understandably, focuses on how biologic therapy may reduce health care costs. This is less clear cut with research using health administrative data failing to demonstrate any significant decrease in hospitalizations or surgical resections in the anti-TNF era compared to what would be expected if these drugs had not been introduced (12). Given this perspective, it is understandable that approaches to reducing the cost of these drugs are being explored.

The emergence of biosimilars, also known as subsequent entry biologics have provided an opportunity for third party payers to reduce anti-TNF therapy drug costs. A biosimilar is a biological medical product that is similar to the original but manufactured by a different company once the patent for that product has expired. They are typically less expensive than the original product and therefore an obvious target in attempts to reduce biologic drug costs. Biosimilars are distinct from usual generic drugs, which are simple small molecules that are relatively straightforward to reproduce and manufacture on a large scale, and identical to the original drug. Biologic therapies are more complex proteins and require replication in living cells. The product is dependent on the type of genetically modified cell being used, the production process and purification techniques (13). The manufacturing process is considerably more expensive than standard small molecules and therefore costs of biosimilars are higher than generically produced drugs. Furthermore, the variation from the originator is greater than would normally be seen with generics. However, it is important to emphasize that even with the original manufacturer there is potential for variability between each manufacturing run due to the complexity of living organisms. The Federal Drug Agency has released documents regarding the approval process for biosimilars (14) that other regulatory authorities have largely adopted (15). Essentially biosimilars must show a high degree of similarity to the original product and have no clinically meaningful differences in safety, purity and potency (14). This is a reasonable definition in principle but the definitions of ‘high degree of similarity’ as well as ‘clinically meaningful differences in safety and potency’ need further clarification in clinical practice.

The Canadian Association of Gastroenterology has previously published a position statement on biosimilars (16) but this was 6 years ago, and more data are now available. Crohn’s and Colitis Canada has published a position statement more recently (17) but the two organizations felt it was of value to release a joint position statement after a full literature review. Several positions statements have been released by various organizations (Table 1) but none has provided an explicit literature search, nor have they assessed the quality of evidence of a defined clinical question according to GRADE criteria (18).

**Table 1.** Position statements reached by other groups on the use of biosimilars

Group	Year	Use in naïve patients	Switch for those already on originator	Automatic substitution
CAG	2013	Await studies	No	No
ECCO	2013	Await studies	No	No
Spanish Society of Gastroenterology	2013	Yes	No	No
Polish National Consultant in Gastroenterology	2014	Yes	No	No
Italian IBD group	2014	Yes	Await studies	No
British Society of Gastroenterology	2015	Yes	Yes	No
NHS Wales	2015	Yes (patient doctor choice)	Yes (patient doctor choice)	No
ESPGHAN	2015	Yes	No	No
Belgian IBD Research Group	2015	Yes	No	No
OAG	2016	No	No	No
ECCO	2017	Yes	Yes (patient and doctor choice)	No
ESPGHAN	2019	Yes	Yes	No
Crohn’s and Colitis Foundation	2019	Yes	Yes (patient doctor choice)	No
CCC	2019	Yes	Yes (patient doctor choice)	No
BC Pharmacare	2019	Yes	Yes	Yes

BC, British Columbia; CAG, Canadian Gastroenterology Association; CCC, Crohn’s Colitis Canada; ECCO, European Crohn’s and Colitis Organization; ESPGHAN, European Society for Pediatric Gastroenterology Hepatology and Nutrition; IBD, Inflammatory bowel disease; NHS, National health service; OAG, Ontario Gastroenterology Association.

This position statement will focus on the data available for the comparison of anti-TNF therapies with their biosimilars as these are the only biosimilars that are currently approved in Canada for IBD. The issues raised in this document are likely to apply to other biologics. The search strategy developed to identify relevant papers for the position statement is given in Supplementary Appendix 1. There are a number of systematic reviews on this topic (19–25), however except for one paper (25), they rely on case series of IBD patients starting a biosimilar or switching to a biosimilar with no comparison to those that are prescribed the originator, so are difficult to interpret. We focused on evidence from randomized controlled trials or cohort studies that compared the biosimilar with the originator. The endpoints we focused on were efficacy, safety and also acceptance by the patient. Although there are randomized, controlled clinical trials comparing originator biologics and biosimilars for the management of rheumatoid arthritis, spondylitis and psoriasis (26–30), we restricted the search to IBD as the efficacy and safety of biosimilars may be different in different diseases. We assessed biosimilars compared to the originator in IBD patients naive to either agent as well as evaluating data relating to switching patients already on an anti-TNF drug to a biosimilar. The reason for this categorization is that when switching drug there is also the added issue of patient intolerance due to a nocebo effect (31).

### Biosimilar Versus Originator Treatment in IBD Patients Naive to Anti-TNF Therapy

We identified one randomized controlled trial (32) that compared the biosimilar CT-P13 with originator infliximab in 220 Crohn’s disease patients who had active disease despite nonbiologic therapy. This was a noninferiority trial with a sample size that had 85% power for a noninferiority margin of −20%. The primary endpoint was a 70-point decrease in Crohn’s Disease Activity Index (CDAI) at week 6 but the trial was continued for 1 year. Patients were randomized 1:1:1:1 with patients in two groups continuing on CT-P13 or infliximab throughout the year whilst those in the other two groups were switched to the other medication at week 30, continuing the alternate medication (CT-P13 or originator) for the rest of the year. GRADE evaluation of this trial identified a low risk of bias. At week 6, 69% of the biosimilar group were in remission compared with 74% of the originator infliximab group (5% difference; 95% confidence intervals [CI] 17% in favour of originator infliximab to 7% in favour of CT-P13). At week 30, clinical remission was seen in 55% of the CT-P13 group compared to 57% of the originator infliximab group (−2%; 95% CI = −15% to +12%). At week 30, there were 6 (5%) treatment-related serious adverse events in the CT-P13 group compared with 9 (8%) in the originator infliximab group. Neutralizing antibodies were similar in the CT-P13 (22 (20%)) and originator infliximab (21 [19%]) groups at week 30. There was one additional biosimilar randomized trial (33), but this did not inform the question of interest as it compared intravenous with subcutaneous CT-P13 and there was no originator comparator.

We identified one cohort study (34) that compared biosimilars with originator infliximab in 5050 Crohn’s patients that were infliximab naive. This study used the Système National des Données de Santé French nationwide health administrative database and the primary outcome was a composite endpoint comprising of death, Crohn’s disease related surgery, all cause hospitalization and switch to another biologic therapy. There were 2499 patients in the CT-P13 group compared with 2551 in the originator infliximab group, studied from 2015 to end of June 2017. There was no difference between the two groups for the primary outcome (hazard ratio [HR] in favour of CT-P13 = 0.92; 95% CI = 0.85 to 0.99). There was no difference between the two groups in terms of Crohn’s disease hospitalization (HR = 1.00; 95% CI = 0.90 to 1.11) or surgery (HR = 1.09; 95% CI = 0.92 to 1.28) or switch to another biologic (HR = 0.93; 0.79 to 1.08). There was also no difference between the groups for serious infections (HR = 0.82; 95% CI = 0.61 to 1.11), tuberculosis (HR = 1.10; 95% CI = 0.36 to 3.34) and solid or hematologic malignancy (HR = 0.66; 95% CI = 0.33 to 1.32). The groups were well balanced for age and sex as well as disease duration and previous medications. However, treatment duration was different given that 65% of the originator infliximab group commenced the drug in 2015 compared with only 28% of the CT-P13 group. The originator infliximab group may therefore have had longer to accrue treatment failure and adverse events, and this may have biased the results in favour of the biosimilar. In addition, treatment strategies have changed over time, such as more recent emphasis on a treat-to-target strategy, which would also favour the biosimilar.

The quality of evidence for starting an infliximab naive patient with active Crohn’s disease on CT-P13 rather than originator infliximab to induce and maintain remission is low according to GRADE criteria (Table 2). The evidence from the cohort study is low and evidence from the randomized trial is downgraded two points for imprecision given that the number of patients evaluated is modest and it is plausible within the 95% confidence intervals of these data that originator infliximab results in a 15% increased remission rate compared to the biosimilar. The quality of evidence for active ulcerative colitis is very low according to GRADE criteria as there are no comparative cohort studies or randomized trials in this disease group, so the data are indirect and relate to the evidence from Crohn’s disease. The evidence for equivalence in safety between CT-P13 and originator infliximab was very low according to GRADE criteria. This mainly relies on one cohort study with a limited number of adverse events (Table 2). There are no data for any anti-TNF biosimilars other than infliximab.

**Table 2.** CT-P13 compared to infliximab originator for active Crohn’s disease naive to anti-TNF therapy: summary of findings table

Certainty assessment							No. of patients		Effect		Certainty	Importance
No. of studies	Study design	Risk of bias	Inconsistency	Indirectness	Imprecision	Other considerations	CT-P13	infliximab originator	Relative (95% CI)	Absolute (95% CI)		
**Crohn’s disease remission (follow up: mean 6 weeks; assessed with: A reduction of CDAI > 70 points)**												
1	Randomised trials	Not serious	Not serious	Not serious	Very serious ^a^	None	77/111 (69.4%)	81/109 (74.3%)	**RR 0.96** (0.76–1.22)	**30 fewer per 1,000** (from 178 fewer to 163 more)	⨁⨁◯◯ LOW	CRITICAL
**Switch to another biologic (follow up: mean 12 months; assessed with: Switch of drug in administrative database)**												
1	Observational studies	Very serious ^b^	Not serious	Not serious	Not serious	None	312/2499 (12.5%)	406/2551 (15.9%)	**HR 0.93** (0.79–1.08)	**10 fewer per 1,000** (from 31 fewer to 12 more)	⨁⨁◯◯ LOW	CRITICAL
**Serious infections (follow up: mean 12 months; assessed with: Administrative database)**												
1	Observational studies	Serious ^b^	Not serious	Not serious	Serious ^c^	None	83/2499 (3.3%)	115/2551 (4.5%)	**HR 0.82** (0.61–1.11)	**8 fewer per 1,000** (from 17 fewer to 5 more)	⨁⨁◯◯ LOW	CRITICAL

CI, confidence interval; HR, hazard ratio explanations; RR, risk ratio.

^a^Possibility that CT-P13 is up to 15% less effective than infliximab originator. This was felt to be an important difference when substituting one drug for a similar drug.

^b^Decision to use CT-P13 was up to patient and clinician.

^c^95% CI include important differences in safety.

In the future, there needs to be randomized controlled trials in ulcerative colitis patients and it is important that cohort studies compare the biosimilar with patients on the originator biologic as without this comparator it is not possible to understand which intervention is the most effective and safe.

### Nonmedical Switch from Originator to Biosimilar Anti-TNF Therapy in IBD Patients

**Figure 1.**
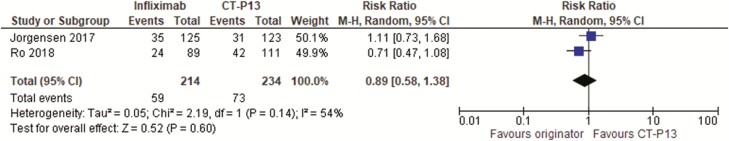
Randomized controlled trials of switch to a biosimilar compared to continuing with originator infliximab in inflammatory bowel disease patients: proportion not in remission at 1 year.

**Figure 2.**
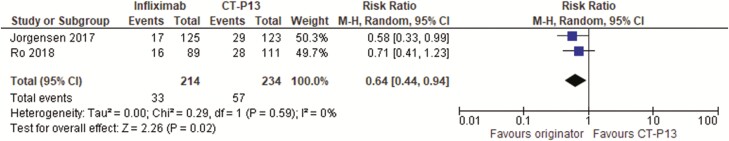
Randomized controlled trials of switch to a biosimilar compared to continuing with originator infliximab in inflammatory bowel disease patients: proportion with loss of response or worsening disease.

We identified two randomized controlled trials (35,36) in 448 IBD patients that were in remission on infliximab for at least 3 (36) to 6 (35) months who were randomized to either continuing on the originator or switching to the biosimilar CT-P13. Both trials followed patients for 1 year and one trial (35) was low risk of bias, whereas the other (36) had unclear method of randomization or concealment of allocation. One trial (35) evaluated patients on biologics for all indications but data on ulcerative colitis and Crohn’s disease could be assessed separately from the information given in the paper and Supplementary Appendix. Overall intention to treat (ITT) remission rates were similar between the two groups with relative risk of not being in remission at one year with infliximab originator = 0.89 (95% CI = 0.58 to 1.38) (Figure 1). There was some heterogeneity between results with an I^2^ of 54% (suggesting that 54% of the variation between the studies was unexplained by chance). However, the ITT loss of response or worsening disease rates were lower with the originator infliximab compared to the biosimilar (relative risk of loss of response/worsening disease = 0.64; 95% CI = 0.44 to 0.94) (Figure 2). There was no heterogeneity between trials with an I^2^ of 0%. The number needed to harm was 11 (95% CI = 6 to 50). One trial (36) only reported data for Crohn’s disease and ulcerative colitis combined whereas the diseases were analysed separately in the other trial (35). This trial (35) suggested that worsening of disease was seen more often with Crohn’s disease (per protocol worsening = −14.3% difference (95% CI = −29.3% to 0.7% in favour of infliximab originator) than with ulcerative colitis (per protocol worsening = −2.6% difference (95% CI = −15.2% to 10.0% in favour of infliximab originator).

Neither randomized trial (35,36) reported safety data specific to IBD patients. One trial (36) did not report on adverse events and the other only gave adverse events for all patients in the trial and therefore included patients with rheumatoid arthritis, ankylosing spondylitis and psoriasis as well as IBD. Serious adverse events were similar between the infliximab originator (24 of 241 [10%] patients) and the CT-P13 (21 of 241 [9%] patients) groups as were overall adverse events (70% versus 68%) and events leading to discontinuation of the drug (4% versus 3%).

We identified two cohort studies (37,38) that assessed 293 IBD patients receiving originator infliximab; overall, 151 patients who continued the originator were compared to 142 patients who switched to the biosimilar (CT-P13). An additional cohort study (39) evaluated patients on infliximab for all indications but did not report results specifically for IBD patients. One of the IBD cohort studies (37) evaluated paediatric patients who either continued on originator infliximab or switched to CT-P13. There was no difference in corticosteroid-free remission rates (defined clinically) without the need for dose intensification; remission was reported in 28/36 (78%) of infliximab continued group and 30/38 (79%) of the CT-P13 switch group (relative risk = 0.99; 95% CI = 0.78 to 1.25). The other IBD cohort study (38) evaluated 219 adults with IBD (120 with Crohn’s disease and 99 with ulcerative colitis) and followed them for one year, evaluating every 4 months with biological markers, the Harvey Bradshaw Index (HBI) for Crohn’s disease and the partial Mayo Score (PMS) for ulcerative colitis. This primary endpoint was disease worsening defined as increase in HBI ≥ 4 or PMS ≥ 5 from baseline. Disease worsening occurred in 8/115 (7%) in the infliximab originator group compared to 11/104 (10.5%) in the CT-P13 switch group (relative risk = 0.66; 95% CI = 0.28 to 1.57). Similarly, the proportion who required biologic dose increase or treatment discontinuation was 16/115 (14%) in the infliximab originator group compared to 22/104 (21%) in the CT-P13 group (relative risk = 0.69; 95% CI = 0.38 to 1.25). Both studies were underpowered and open to bias as the decision to switch was made by the patient and clinician. Nevertheless, the results are similar to the randomized controlled trials in that there was no difference in remission rates but there was a suggestion of a greater proportion of CT-P13 switch patients who deteriorated, lost response or needed a change in therapy compared with those continued originator infliximab therapy. This is supported by another cohort study (39) evaluating all patients on biologic therapy, regardless of indication. This study reported on 1388 patients who continued originator infliximab therapy with 136 patients who switched to CT-P13. Patients who switched to CT-P13 were more likely to discontinue treatment (hazard ratio = 5.53; 95% CI = 4.01 to 7.63) compared to those who continued with originator infliximab therapy for 12 months.

**Figure 3.**
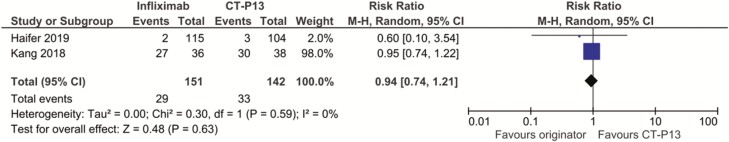
Adverse events in cohort studies of switch to a biosimilar compared to continuing with originator infliximab in inflammatory bowel disease patients.

Both cohort studies reported adverse events, one reporting all adverse events (37) and the other reporting serious adverse events (38). Both studies found no difference in adverse events between groups and the overall relative risk of adverse events in the infliximab originator group was 0.94 (95% CI = 0.74 to 1.21; Figure 3). The cohort study (39) assessing those on infliximab for all indications also found no difference in adverse events between those continuing on infliximab originator compared to CT-P13 (adjusted incidence ratio = 0.67; 95% CI = 0.19 to 2.30).

In summary, the evidence is against switching from originator infliximab to CT-P13 in IBD patients who are doing well on the original drug. This is very low-quality evidence according to GRADE criteria based on randomized trial evidence that patients on CT-P13 have a higher risk of worsening of disease and need to dose escalate or switch (Table 3). The evidence was downgraded as one study had unclear risk of bias and it was downgraded two further levels for imprecision and indirectness. There is evidence that both drugs have a similar safety profile, but this is again very low-quality evidence according to GRADE criteria (Table 3).

**Table 3.** Nonmedical switching to CT-P13 compared to continuing on infliximab originator for stable IBD on originator infliximab: summary of findings table

Certainty assessment							No. of patients		Effect		Certainty	Importance
No. of studies	Study design	Risk of bias	Inconsistency	Indirectness	Imprecision	Other considerations	CT-P13	infliximab originator	Relative (95% CI)	Absolute (95% CI)		
**Not in remission (follow up: mean 12 months; assessed with: Defined clinically)**												
2	Randomised trials	Serious ^a^	Not serious	Not serious	Serious ^b^	None	73/234 (31.2%)	59/214 (27.6%)	**RR 0.89** (0.58–1.38)	**30 fewer per 1,000** (from 116 fewer to 105 more)	⨁⨁◯◯ LOW	CRITICAL
**loss of response or worsening of disease (follow up: mean 12 months; assessed with: clinical opinion)**												
2	randomised trials	serious ^a^	not serious	serious ^c^	serious ^b^	none	57/234 (24.4%)	33/214 (15.4%)	**RR 0.64** (0.44–0.94)	**56 fewer per 1,000** (from 86 fewer to 9 fewer)	⨁◯◯◯ VERY LOW	CRITICAL
**Adverse events (follow up: mean 12 months; assessed with: Assessed clinically (either all adverse events or serious adverse events))**												
2	Observational studies	Serious ^d^	Not serious	Serious ^c^	Serious ^b^	None	33/142 (23.2%)	29/151 (19.2%)	**RR 0.94** (0.74–1.21)	**12 fewer per 1,000** (from 50 fewer to 40 more)	⨁◯◯◯ VERY LOW	CRITICAL

CI, confidence interval; RR, risk ratio.

^a^One of the two trials had an unclear risk of bias.

^b^95% CI wide with number of events < 250.

^c^Each trial defined loss of response differently.

^d^Two observational studies where decision to switch to CT-P13 is up to patient and clinician.

Future studies should focus on comparative cohort studies comparing the originator biologic with its biosimilar and not simply providing information on experience with the biosimilar. Without comparative data, it is not possible to reach evidence-based conclusions.

### Cost-effectiveness of Using Biosimilars Compared to the Originator Anti-TNF

The Canadian cost of originator infliximab (Remicade) is $4,471 CAD compared with $1,934 CAD for CT-P13 (Inflectra) per claim. These costs are derived from aggregate claims data from the National Prescription Drug Utilization Information System (40). This source does not include undisclosed discounts and rebates that are in place for both products and so actual costs are difficult to determine as there are no publicly available data of the actual cost to the payer. We identified one health economic model (41) that compared infliximab originator versus the biosimilar for active Crohn’s disease using UK costs over a 1 year time frame. This model found biosimilar therapy would be more cost-effective approximately 85% of the time in a probabilistic analysis based on the model’s assumptions that £30,000 per quality adjusted life year gained was the threshold for cost-effectiveness. This model only evaluated biologic-naive patients started on infliximab originator or the biosimilar and assumed that both were equally effective with no uncertainty in the model for efficacy. The main area of uncertainty addressed in the model was differences in anti-drug antibody development. While this is one area of uncertainty there are others and in particular the model could have used the −2% (95% CI = −15% to +12%) difference in efficacy from the randomized trial in biologic naive Crohn’s disease patients.

We did not identify any health economic models that evaluated switching to a biosimilar in IBD patients who achieved remission on the originator biologic. However, one cohort study (39) evaluated health care costs in those remaining on infliximab originator for any indication versus those switched to CT-P13. This Turkish cohort study (39) found that those remaining on the originator cost less than those switching to CT-P13 (average total health care costs = 2009 Turkish Lira per patient in the CT-P13 group compared to 1640 Turkish Lira in the originator group, *P* = 0.046). This is consistent with a systematic review (42) of nonmedical switching of all types of drugs on economic outcomes which found that, despite the generic being cheaper, overall health care costs increased in 69% of studies and were neutral in 31% with no study reporting a cost saving (42).

### Recommendations

We suggest that an infliximab biosimilar may be started in patients with active Crohn’s disease who are naive to anti-TNF therapy rather than starting with the infliximab originator. This is a weak recommendation based on low-quality evidence and depends on the price differential of the two drugs. If the price differential is modest, then the infliximab originator should be used. However, if the price differential is comparable to that calculated based on the current, published list price of the drugs, it is plausible that the biosimilar is more cost-effective. The weak recommendation implies that the clinician should discuss risks and benefits carefully with the patient, taking into account the preference of the patient, and decisions should be made on a case-by-case basis. There are insufficient data to recommend the use of biosimilars in patients with active ulcerative colitis naive to infliximab.

We recommend against nonmedical switching from originator infliximab to biosimilar in patients who have stable IBD and are doing well on the original product. This is a weak recommendation based on very low-quality evidence but data suggest that switching in this setting leads to an increased risk of worsening of disease, dose escalation and/or switching to an alternative therapy.

We do not recommend automatic substitution of biologic with a biosimilar in IBD patients given the paucity of evidence for the efficacy and safety of this approach.

## Conflict of Interest

P.M., E.I.B., C.Y., A.F., and G.I.L. declared that, over the last 2 years, they had no conflicts relevant to the topics of this Position Statement. D.A. declared that, over the last 2 years, he received honoraria from Takeda (a pharmaceutical company producing biologics) for participating in advisory boards that discussed vedolizumab and teduglutide, neither of which was related to the topics of this Position Statement (vedolizumab is not an anti-TNF biologic and, furthermore, it is protected by patents such that no relevant biosimilars can be produced currently; teduglutide is not a biologic), and that he received a research grant from ABBVIE (a pharmaceutical company producing biologics) for an investigator-initiated study (‘Inflammation-related differences in mucosa-associated microbiota and intestinal barrier function in colonic Crohn’s disease’) that is not related to the topics of this Position Statement. P.M. is Nominated Principle Applicant, AF is Executive Director and all authors are involved with the ‘Inflammation, Microbiome, and Alimentation: Gastro-Intestinal and Neuropsychiatric Effects: the IMAGINE network’—a Strategy for Patient-Oriented Research CIHR Chronic Disease Network. The research conducted by this network has no hypothesis that is directly related to biologics other than evaluation of how the microbiome and diet may allow more precise targeting of patients with inflammatory bowel disease who are likely to respond to treatment.

## Supplementary Material

gwz035_suppl_Supplementary_Appendix-1

## References

[1] Ford AC , Sandborn WJ, Khan KJ, et al. Efficacy of biological therapies in inflammatory bowel disease: Systematic review and meta-analysis. Am J Gastroenterol 2011;106(4):644–59, quiz 660. 21407183 10.1038/ajg.2011.73

[2] Frolkis AD , Dykeman J, Negrón ME, et al. Risk of surgery for inflammatory bowel diseases has decreased over time: A systematic review and meta-analysis of population-based studies. Gastroenterology 2013;145(5):996–1006. 23896172 10.1053/j.gastro.2013.07.041

[3] LeBlanc K , Mosli MH, Parker CE, MacDonald JK. The impact of biological interventions for ulcerative colitis on health-related quality of life. Cochrane Database Syst Rev 2015, Issue 9. Art. No.: CD008655. doi:10.1002/14651858.CD008655.pub3 PMC9235035 26393522

[4] Fiorino G , Danese S, Pariente B, et al. Paradoxical immune-mediated inflammation in inflammatory bowel disease patients receiving anti-TNF-α agents. Autoimmun Rev 2014;13(1):15–9. 23777821 10.1016/j.autrev.2013.06.005

[5] Vande Casteele N , Khanna R, Levesque BG, et al. The relationship between infliximab concentrations, antibodies to infliximab and disease activity in Crohn’s disease. Gut 2015;64(10):1539–45. 25336114 10.1136/gutjnl-2014-307883 PMC4602247

[6] Shah ED , Farida JP, Siegel CA, et al. Risk for overall infection with anti-TNF and anti-integrin agents used in IBD: A systematic review and meta-analysis. Inflamm Bowel Dis 2017;23(4):570–7. 28230558 10.1097/MIB.0000000000001049

[7] Targownik LE , Bernstein CN. Infectious and malignant complications of TNF inhibitor therapy in IBD. Am J Gastroenterol 2013;108(12):1835–42, quiz 1843. 24042192 10.1038/ajg.2013.294

[8] https://www.cihi.ca/en/health-spending/2018/prescribed-drug-spending-in-canada (Accessed October 22, 2019).

[9] Kuenzig ME , Lee L, El-Matary W, et al. The impact of inflammatory bowel disease in Canada 2018: Indirect costs of IBD care. J Can Assoc Gastroenterol 2019;2(Suppl 1):34–41. 10.1093/jcag/gwy050 PMC6512236 31294383

[10] https://crohnsandcolitis.ca/Crohns_and_Colitis/documents/reports/2018-Impact-Report-LR.pdf

[11] Holko P , Kawalec P, Pilc A. Cost-effectiveness analysis of Crohn’s disease treatment with vedolizumab and ustekinumab after failure of tumor necrosis factor-α antagonist. Pharmacoeconomics 2018;36(7):853–65. 29667146 10.1007/s40273-018-0653-2 PMC5999163

[12] Murthy SJ , Begum J, Benchimol EI, et al. Introduction of anti-TNF therapy has not yielded expected declines in hospitalisations and intestinal resection rates in inflammatory bowel diseases: A population-based interrupted time series study. Gut 2019. doi:10.1136/gutjnl-2019-318440 PMC6984056 31196874

[13] Weise M , Kurki P, Wolff-Holz E, et al. Biosimilars: The science of extrapolation. Blood 2014;124(22):3191–6. 25298038 10.1182/blood-2014-06-583617

[14] https://www.fda.gov/drugs/biosimilars/biosimilar-development-review-and-approval.

[15] https://www.canada.ca/en/health-canada/services/drugs-health-products/biologics-radiopharmaceuticals-genetic-therapies/applications-submissions/guidance-documents/fact-sheet-biosimilars.html

[16] Devlin SM , Bressler B, Bernstein CN, et al. Overview of subsequent entry biologics for the management of inflammatory bowel disease and Canadian Association of Gastroenterology position statement on subsequent entry biologics. Can J Gastroenterol 2013;27(10):567–71. 24106727 10.1155/2013/327120 PMC3805336

[17] http://www.crohnsandcolitis.ca/Crohns_and_Colitis/documents/2019-September-CCC-Biosimilars-Position-Statement.pdf

[18] Guyatt GH , Oxman AD, Vist GE, et al.; GRADE Working Group. GRADE: An emerging consensus on rating quality of evidence and strength of recommendations. BMJ 2008;336(7650):924–6. 18436948 10.1136/bmj.39489.470347.AD PMC2335261

[19] Radin M , Sciascia S, Roccatello D, et al. Infliximab biosimilars in the treatment of inflammatory bowel diseases: A systematic review. BioDrugs 2017;31(1):37–49. 28035633 10.1007/s40259-016-0206-1

[20] Komaki Y , Yamada A, Komaki F, et al. Systematic review with meta-analysis: The efficacy and safety of CT-P13, a biosimilar of anti-tumour necrosis factor-α agent (infliximab), in inflammatory bowel diseases. Aliment Pharmacol Ther 2017;45(8):1043–57. 28239873 10.1111/apt.13990

[21] Milassin Á , Fábián A, Molnár T. Switching from infliximab to biosimilar in inflammatory bowel disease: Overview of the literature and perspective. Therap Adv Gastroenterol 2019;12:1756284819842748. 10.1177/1756284819842748 PMC6469269 31019554

[22] Ebada MA , Elmatboly AM, Ali AS, et al. An updated systematic review and meta-analysis about the safety and efficacy of infliximab biosimilar, CT-P13, for patients with inflammatory bowel disease. Int J Colorectal Dis 2019;34(10):1633–52. 31492986 10.1007/s00384-019-03354-7

[23] Kashani A , Syal G, Bonthala N, McGovern DPB, David S. Efficacy of infliximab biosimilar for induction and maintenance therapy in inflammatory bowel disease after switch from drug originator: A meta-analysis (abstract). Am J Gastroenterol 2017;112(supplement 1):S390, abstract 705.

[24] Martelli L , Peyrin-Biroulet L. Efficacy, safety and immunogenicity of biosimilars in inflammatory bowel diseases: A systematic review. Curr Med Chem 2019;26(2):270–9. 27758715 10.2174/0929867323666161014153346

[25] Feagan BG , Lam G, Ma C, et al. Systematic review: Efficacy and safety of switching patients between reference and biosimilar infliximab. Aliment Pharmacol Ther 2019;49(1):31–40. 30411382 10.1111/apt.14997 PMC6587715

[26] Smolen JS , Choe JY, Prodanovic N, et al. Safety, immunogenicity and efficacy after switching from reference infliximab to biosimilar SB2 compared with continuing reference infliximab and SB2 in patients with rheumatoid arthritis: Results of a randomised, double‐ blind, phase III transition study. Ann Rheum Dis. 2018;77:234–240. 29042358 10.1136/annrheumdis-2017-211741 PMC5867419

[27] Park W , Hrycaj P, Jeka S, et al. A randomised, double-blind, multicentre, parallel-group, prospective study comparing the pharmacokinetics, safety, and efficacy of CT-P13 and innovator infliximab in patients with ankylosing spondylitis: The PLANETAS study. Ann Rheum Dis 2013;72(10):1605–12. 23687259 10.1136/annrheumdis-2012-203091 PMC3786643

[28] Yoo DH , Hrycaj P, Miranda P, et al. A randomised, double-blind, parallel-group study to demonstrate equivalence in efficacy and safety of CT-P13 compared with innovator infliximab when coadministered with methotrexate in patients with active rheumatoid arthritis: The PLANETRA study. Ann Rheum Dis 2013;72(10):1613–20. 23687260 10.1136/annrheumdis-2012-203090 PMC3786641

[29] Choe JY , Prodanovic N, Niebrzydowski J, et al. A randomised, double-blind, phase III study comparing SB2, an infliximab biosimilar, to the infliximab reference product Remicade in patients with moderate to severe rheumatoid arthritis despite methotrexate therapy. Ann Rheum Dis 2017;76(1):58–64. 26318384 10.1136/annrheumdis-2015-207764 PMC5264229

[30] Cohen SB , Alten R, Kameda H, et al. A randomized controlled trial comparing PF-06438179/GP1111 (an infliximab biosimilar) and infliximab reference product for treatment of moderate to severe active rheumatoid arthritis despite methotrexate therapy. Arthritis Res Ther 2018;20(1):155. 30053896 10.1186/s13075-018-1646-4 PMC6063022

[31] Straka RJ , Keohane DJ, Liu LZ. Potential clinical and economic impact of switching branded medications to generics. Am J Ther 2017;24(3):e278–89. 26099048 10.1097/MJT.0000000000000282 PMC5417581

[32] Ye BD , Pesegova M, Alexeeva O, et al. Efficacy and safety of biosimilar CT-P13 compared with originator infliximab in patients with active Crohn’s disease: An international, randomised, double-blind, phase 3 non-inferiority study. Lancet 2019;393(10182):1699–707. 30929895 10.1016/S0140-6736(18)32196-2

[33] Ye BD SC , Jang BI, Borzan V, Lahat A, Pukitis A, et al. A novel forumaltion of CT-P13 (infliximab biosimilar) for subcutaneous administration 1-year result from a phase 1 open-label randomized controlled trial in patients with active Crohn’s disease (abstract). Gastroenterology 2019;156(supplement 1):S1096.

[34] Meyer A , Rudant J, Drouin J, et al. Effectiveness and safety of reference infliximab and biosimilar in Crohn disease: A French equivalence study. Ann Intern Med 2019;170(2):99–107. 30534946 10.7326/M18-1512

[35] Jørgensen KK , Olsen IC, Goll GL, et al.; NOR-SWITCH study group. Switching from originator infliximab to biosimilar CT-P13 compared with maintained treatment with originator infliximab (NOR-SWITCH): A 52-week, randomised, double-blind, non-inferiority trial. Lancet 2017;389(10086):2304–16. 28502609 10.1016/S0140-6736(17)30068-5

[36] Ro der H , Schnigzler F, Borchardt J, Janelidze S, Ochsenkuhn T. Switch of infliximab originator to biosimilar CT-P13 in patients with Crohn’s disease and ulcerative colitis in a large German IBD center. A one year, randomized and prospective trial (abstract). United European Gastroenterol J 2018;8(Supplement):A456.

[37] Kang B , Lee Y, Lee K, et al. Long-term outcomes after switching to CT-P13 in pediatric-onset inflammatory bowel disease: A single-center prospective observational study. Inflamm Bowel Dis 2018;24(3):607–16. 29390113 10.1093/ibd/izx047

[38] Haifer C , Srinivasan A, Menon S, et al. Switching Australian patients with moderate to severe inflammatory bowel disease from originator infliximab to biosimilar Inflectra: Interim results from a multicenter parallel cohort study (abstract). J Gastroenterol Hepatol 2019;34(supplement 2):155–6.

[39] Phillips K , Juday T, Zhang Q, Keshishian A. Economic outcomes, treatment patterns, and adverse events and reactions for patients prescribed infliximab or CT-P13 in the Turkish population. Ann Rheum Dis 2017;76(supplement 2):835.

[40] Moura CS , Choquette D, Boire G, et al. Inflectra and Remicade use and cost in Canada under provincial drug plans in 2016 (abstract). Arthritis Rheum 2018;70(supplement 10):Abstract 216.

[41] Catt H , Bodger K, Kirkham JJ, Hughes DA. Value assessment and quantitative risk-benefit modelling of biosimilar infliximab for Crohn’s disease. PharmacoEconomics 2019. doi:10.1007/s40273-019-00826 31372948

[42] Nguyen E , Weeda ER, Sobieraj DM, et al. Impact of non-medical switching on clinical and economic outcomes, resource utilization and medication-taking behavior: A systematic literature review. Curr Med Res Opin 2016;32(7):1281–90. 27033747 10.1185/03007995.2016.1170673

