# Supplementary material for: Joint Canadian Association of Gastroenterology and Crohn’s Colitis Canada Position Statement on Biosimilars for the Treatment of Inflammatory Bowel Disease
Source: J Can Assoc Gastroenterol. 2019 Nov 8;3(1):e1–9. doi: 10.1093/jcag/gwz035 (PMC6985688; doi:10.1093/jcag/gwz035)
Supplement: gwz035_suppl_Supplementary_Appendix-1 [file gwz035_suppl_supplementary_appendix-1.docx]

**Appendix: Search strategy.**

Database: OVID Medline Epub Ahead of Print, In-Process & Other Non-Indexed Citations, Ovid MEDLINE(R) Daily and Ovid MEDLINE(R) 1946 to Present, Embase <1974 to 2019 October 08>, EBM Reviews - Cochrane Central Register of Controlled Trials <September 2019>, EBM Reviews - Cochrane Database of Systematic Reviews <2005 to October 3, 2019>

Search Strategy:

--------------------------------------------------------------------------------

1 (inflammatory bowel disease* or ulcerative colitis or Crohn* or IBD).tw,kw. (260985)

2 exp Biosimilar Pharmaceuticals/ (5959)

3 exp biosimilar agent/ (4133)

4 (biosimilar* or inflectra).tw,kw. (10755)

5 (biologic* adj3 (follow-on or subsequent entry or similar)).tw,kw. (9120)

6 or/2-5 (20290)

7 1 and 6 (1358)

8 randomized controlled trial.pt. (971168)

9 clinical trial.pt. (797929)

10 random*.mp. (3989679)

11 placebo.ab. (752778)

12 drug therapy.fs. (5781246)

13 (placebo: or double-blind:).mp. (1248843)

14 clinical trial:.mp. (3082388)

15 blind:.tw. (997409)

16 or/8-15 (10559041)

17 7 and 16 (891)

18 controlled study/ or major clinical study/ (8856293)

19 comparative study/ or (comparative or comparison or compared or controlled).tw. (13227283)

20 exp cohort analysis/ or exp Cohort Studies/ (2569068)

21 (cohort or cohorts).tw,kw. (1522008)

22 prospective*.tw,kw. (1882262)

23 (followed or follow up).tw. (4564759)

24 exp longitudinal study/ or exp Longitudinal Studies/ (395029)

25 (longitudinal or time series).tw. (614437)

26 ((evaluat* or multicenter) adj3 (studies or study)).tw. (1373139)

27 ((population or hospital) adj based).tw. (352933)

28 (trial or groups).ab. (6264896)

29 or/18-28 (25200684)

30 7 and 29 (950)

31 17 or 30 (1227)

32 (exp animals/ or exp animal/ or exp nonhuman/ or exp animal experiment/ or animal model/ or animal tissue/ or non human/ or (rat or rats or mice or mouse or swine or porcine or murine or sheep or lambs or pigs or piglets or rabbit or rabbits or cat or cats or dog or dogs or cattle or bovine or monkey or monkeys or trout or marmoset$).ti.) not (humans/ or human/ or human experiment/ or (human* or men or women or patients or subjects).tw.) (10170739)

33 31 not 32 (1224)

34 remove duplicates from 33 (867)

35 limit 34 to english language [Limit not valid in CDSR; records were retained] (847)
